# The biological and immunological significance of the estrogen-related gene IER3 in diabetes

**DOI:** 10.3389/fendo.2025.1570332

**Published:** 2025-09-02

**Authors:** Da Ke, Xian He, Wenzhe Li, Hongyan Wu, Yaling Sun, Jie Tan, Ya Wang

**Affiliations:** ^1^ Department of Endocrinology, The First Affiliated Hospital of Yangtze University, Jingzhou First People’s Hospital, Jingzhou, Hubei, China; ^2^ Department of Hubei Provincial Clinical Research Center for Personalized Diagnosis and Treatment of Cancer, The First Affiliated Hospital of Yangtze University, Jingzhou First People’s Hospital, Jingzhou, Hubei, China; ^3^ Department of Hematology, The First Affiliated Hospital of Yangtze University, Jingzhou First People’s Hospital, Jingzhou, Hubei, China

**Keywords:** diabetes mellitus, glycometabolism, estrogen, bioinformatics analysis, machine learning, IER3

## Abstract

**Background:**

Diabetes Mellitus (DM) is a complex metabolic disorder characterized by hyperglycemia, primarily arising from insufficient insulin secretion or the development of insulin resistance. Estrogen plays a significant role in regulating the occurrence and progression of DM. This study aims to investigate the role of estrogen-related genes in diabetes, focusing on identifying potential biomarkers and therapeutic targets for the disease.

**Methods:**

We initially obtained gene expression datasets related to type 2 diabetes mellitus (T2DM) from the GEO database. A systematic and coherent series of methodologies was then implemented in a structured manner. First, Principal Component Analysis (PCA) was employed for preliminary data exploration and dimensionality reduction. Next, we identified Differentially Expressed Genes (DEGs). Subsequently, we conducted Weighted Gene Co-expression Network Analysis (WGCNA) to uncover gene modules associated with DM. This was followed by Gene Ontology (GO) and Kyoto Encyclopedia of Genes and Genomes (KEGG) enrichment analyses to explore the biological functions and pathways associated with the identified genes. To enhance the precision of biomarker identification, we applied three distinct machine learning algorithms, including Least Absolute Shrinkage and Selection Operator (LASSO), Support Vector Machine-Recursive Feature Elimination (SVM-RFE), and Random Forest (RF), for further refined selection. This comprehensive approach ultimately identified the estrogen-related gene IER3 as a promising biomarker for DM. Furthermore, correlation analyses focusing on immune cell infiltration were conducted to clarify the immunological role of IER3 in DM.

**Results:**

Our findings revealed a significant downregulation of IER3 in DM patients, accompanied by an AUC value of 0.723 in the diagnostic curve ROC, indicating its considerable diagnostic and prognostic potential for DM. Furthermore, the expression levels of IER3 exhibited a strong correlation with variations in the proportions of diverse immune cell types, suggesting that it may play a pivotal role in the immunoregulatory mechanisms underlying DM.

**Conclusion:**

In conclusion, our findings reveal that the estrogen-related gene IER3 is significantly downregulated in patients with DM, highlighting its potential as a diagnostic and prognostic marker for the disease. Therefore, IER3 may serve as a promising biomarker and therapeutic target for DM.

## Graphical Abstract

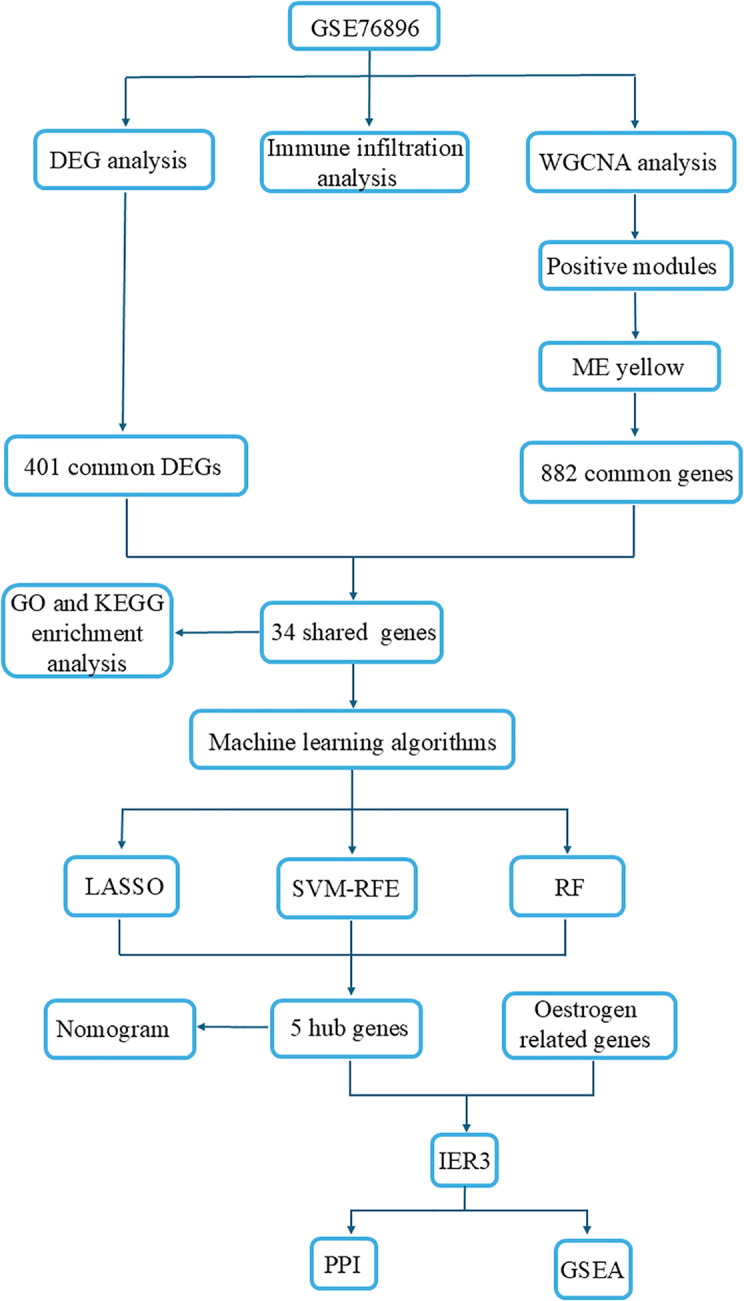

## 1 Introduction

Diabetes mellitus (DM) is characterized by hyperglycemia and encompasses several types, primarily type 1 diabetes mellitus (T1DM), type 2 diabetes mellitus (T2DM), and gestational diabetes mellitus (GDM). The primary pathological mechanisms underlying DM involve either inadequate insulin secretion or the presence of insulin resistance, resulting in sustained elevations in blood glucose levels (1, 2). This hyperglycemic state not only disrupts systemic metabolism but also inflicts damage to multiple organs and systems. Chronic hyperglycemia is a contributing factor to both microvascular and macrovascular complications, leading to conditions such as diabetic retinopathy, diabetic nephropathy, diabetic neuropathy, alongside a variety of gynecological malignancies (3, 4). Furthermore, individuals with DM demonstrate a markedly higher incidence of cardiovascular diseases, contributing to a cardiovascular mortality rate that exceeds that of individuals without DM (5, 6). Preventive strategies for DM emphasize the importance of managing established risk factors, including obesity, hypertension, and unhealthy dietary habits, while also promoting public awareness of DM through health policies designed to enhance early screening rates. Notably, early intervention in T2DM has been shown to effectively delay or prevent the onset of the disease.

Estrogens, a class of steroid hormones predominantly secreted by the ovaries, include estradiol (E2), estrone (E1), and estriol (E3). These hormones play a crucial role in the development of the female reproductive system, the manifestation of secondary sexual characteristics, and a multitude of physiological functions (7). Recent advancements in understanding of estrogen signaling mechanisms have yielded a more nuanced perspective on their roles in various physiological processes. Within the female reproductive system, estrogens are primarily responsible for promoting the development and maturation of ovarian follicles, sustaining endometrial proliferation, and facilitating ovulation. Additionally, estrogens have garnered considerable attention for their protective effects on bone health, as they help maintain bone density by promoting bone matrix synthesis and inhibiting bone resorption, thereby effectively reducing the risk of osteoporosis in postmenopausal women (8). Furthermore, estrogens exert significant influences on cognitive function, mood regulation, and neuroprotection, with clinical studies suggesting their positive impact on slowing the progression of Alzheimer’s disease (9).

It is essential to highlight the significant role that estrogens play in DM. At certain concentrations, elevated estrogen levels can enhance insulin sensitivity, thereby reducing the risk of developing DM (10). Specifically, estrogens exert their effects by binding to specific receptors and activating signaling pathways such as PI3K/Akt and MAPK, which subsequently influence both insulin secretion and action (11). This interaction ultimately modulates the onset and progression of DM (12, 13). Given the intricate interplay between estrogens and DM, alongside the current gaps in understanding their molecular mechanisms and pathological interactions, recent advancements in biotechnology offer valuable tools for exploring the underlying mechanisms linking these two factors.

This study utilizes a comprehensive bioinformatics approach combined with machine learning techniques to investigate the shared genes and associated signaling pathways linking estrogens and DM. By elucidating the specific pathogenic mechanisms of estrogen-related genes in the context of DM, this research offers valuable data support and identifies potential breakthroughs for more targeted and effective prevention and treatment strategies for DM.

## 2 Materials and methods

### 2.1 Data acquisition and preprocessing

**Graphical Abstract** illustrates the workflow of this study. The gene expression dataset for DM was sourced from the GEO database (https://www.ncbi.nlm.nih.gov/geo/) using “diabetes” as the search term. We applied filtering criteria including “DataSets Database” and “Homo sapiens” to refine the dataset. Specimens related to “methylation,” “diabetic nephropathy,” and “non-pancreatic tissues” were excluded from consideration. Ultimately, we selected sequencing data from the T2DM group and the normal pancreatic tissue group for further analysis. Based on the aforementioned selection criteria, GSE76896 was identified as the discovery cohort, comprising a total of 206 samples, including 117 from the normal group, 55 from the T2DM group, while 34 samples from the impaired glucose tolerance group were excluded.

### 2.2 Principal component analysis

To reduce dimensionality and facilitate the visualization of sample clustering, PCA was conducted on the original dataset, with all preprocessing executed utilizing the “affy” package in R (14). Probes were converted to gene symbols based on the GPL570 platform (Affymetrix Human Genome U133 Plus 2.0 Array). PCA serves as a dimensionality reduction technique that applies orthogonal transformation to reconfigure the data into a new coordinate system, thereby maximizing variance along these new axes. This approach preserves the most significant features of the data and enables visualization of the distribution of high-dimensional data across the first two principal components.

### 2.3 Identification of differentially expressed genes in DM

We utilized the “Limma” package in R to identify DEGs within the GSE76896 dataset. The criteria for DEG selection were established as an adjusted p-value of <0.05 and a log-fold change (logFC) of ≥0.70. Additionally, we constructed a volcano plot to visually depict the statistical significance and magnitude of expression changes associated with these DEGs. This approach enables researchers to effectively identify target genes that exhibit significant upregulation or downregulation under disease conditions.

### 2.4 Weighted gene co-expression network analysis and module gene identification

We employed the R package “WGCNA” to identify biologically meaningful co-expression gene modules and to explore the relationship between gene networks and disease (15). Initially, the top 10,000 genes with the highest variance were selected for further analysis. Subsequently, the “pickSoft Threshold” function was utilized to determine the optimal soft thresholding power (β), which ranges from 1 to 20, in order to construct a scale-free network. The average connectivity R² threshold was set at 0.85. Following this, the adjacency matrix was transformed into a Topological Overlap Matrix (TOM) to evaluate gene ratios and dissimilarity. In the fourth step, hierarchical clustering and the dynamic tree cut function were applied to delineate and identify co-expression modules. These modules were then merged based on analogous expression patterns for further analysis, with the parameters “minModuleSize” and “deepSplit” set to 150 and 2, respectively. In the fifth step, we examined the correlation between modules and disease by calculating Gene Significance (GS) and Module Membership (MM). Genes within the modules that exhibited the strongest correlation with the disease were selected for further investigation. Finally, we conducted an intersection analysis between the DEGs and the genes identified through WGCNA, which yielded a set of 34 common genes. We visualized these shared genes using clustering heatmaps generated by the “ggplot2” and “pheatmap” R packages (16). This step aims to identify co-expression modules that are significantly associated with DM, thereby providing a candidate set of genes for subsequent functional enrichment analysis and machine learning screening.

### 2.5 Functional enrichment analysis

To further investigate the biological functions and signaling pathway characteristics of diabetes-related genes, as well as to elucidate their potential molecular mechanisms, we conducted functional enrichment analysis using the “clusterProfiler” and “ggplot2” R packages. This approach facilitated an efficient evaluation and visualization of gene functionality. In the Gene Ontology (GO) analysis, genes were categorized into three main functional categories: Biological Process (BP), Cellular Component (CC), and Molecular Function (MF). This categorization enhances our comprehension of the roles of genes across various biological dimensions. Additionally, Kyoto Encyclopedia of Genes and Genomes (KEGG) pathway enrichment analysis offers a systematic framework for investigating gene functions, particularly concerning cellular signaling and metabolic pathways. To ensure the statistical significance of the analysis results, we established a cutoff criterion for p-values and q-values at 0.05.

### 2.6 Machine learning approaches for identifying candidate biomarkers

To accurately identify candidate biomarkers associated DM from extensive genomic datasets, we employed machine learning methodologies. These algorithms have gained prominence in the field of bioinformatics due to their robust capabilities for handling complex datasets (17). They are capable of extracting critical information from gene expression data and identifying the genes that are most pertinent to specific disease states. By leveraging machine learning techniques, we can more effectively manage high-dimensional data, uncover nonlinear relationships, and filter potential biomarkers. This approach enhances predictive accuracy and addresses challenges that frequently confound traditional statistical methods. Consequently, machine learning was used in this study to further refine candidate genes with the aim of discovering novel biomarkers for DM. We employed three widely recognized machine learning algorithms to further refine the selection of candidate biomarkers: Least Absolute Shrinkage and Selection Operator (LASSO) (18), Support Vector Machine-Recursive Feature Elimination (SVM-RFE) (19), and Random Forest (RF) (20). LASSO is a regularized regression technique that applies an L1 penalty to shrink the coefficients of less informative variables to zero, thus facilitating simultaneous variable selection and regularization. SVM-RFE is a backward feature elimination method based on support vector machines, which recursively eliminates features with the lowest ranking weights to identify the subset that optimally separates the classes. RF, an ensemble learning approach based on decision trees, trains each tree on a bootstrap sample and a subset of features, allowing for the assessment of feature importance via the mean decrease in impurity. These three algorithms collectively enhance the feature selection process: LASSO prioritizes sparsity, SVM-RFE focuses on margin-based discrimination, and RF utilizes ensemble-based ranking. This complementary synergy significantly bolsters the robustness and reliability of the selected biomarkers. Candidate genes identified through the intersection of these algorithms were considered highly reliable for subsequent analysis.

### 2.7 Expression analysis and diagnostic evaluation of candidate genes for DM

To further verify the diagnostic efficacy of candidate genes and construct a clinically applicable risk assessment model, the “ggplot2” package was utilized to assess the expression levels of candidate biomarkers in both control and DM groups, with a significance threshold set at p < 0.05. A Nomogram was constructed using the “rms” package, wherein “Points” represent the scores assigned to the candidate genes, and the “Total Score” denotes the cumulative score across all the aforementioned genes. To evaluate the diagnostic accuracy of the candidate biomarkers, the area under the receiver operating characteristic (ROC) curve (AUC) was calculated using the “pROC” package.

### 2.8 Identification of candidate biomarkers

Candidate genes related to estrogen were retrieved from the NCBI (National Center for Biotechnology Information, https://www.ncbi.nlm.nih.gov/gene) database using the search terms “oestrogen” and “Homo sapiens”. These estrogen-related genes were subsequently intersected with genes linked to DM, with selection criteria requiring an AUC ≥ 0.7 for further analysis. After screening and identifying five candidate genes, we conducted a comprehensive evaluation of each and determined that IER3 exhibits the highest research value for the following reasons:A. Estrogen linkage: Previous studies have demonstrated that OHT, a related compound, stimulates IER3 expression in an estrogen receptor-dependent manner (21). In contrast, other genes, including LRRK2, have not shown a similar association.B. Immune modulation: IER3 is a well-established immunoregulatory gene. For instance, induction of IER3 protects macrophages from LPS-induced apoptosis and inhibits NF-κB activity (22). This function in modulating inflammation is directly relevant to diabetes, which is characterized by chronic immune dysregulation.C. Metabolic inflammation: IER3 plays a crucial role in mediating metabolic and immune crosstalk in obesity. Mice deficient in IER3 exhibit reduced adipose inflammation and improved insulin sensitivity under high-fat diet conditions (23). This demonstrates that IER3 plays a significant role in regulating the interface between metabolism and immune responses.

### 2.9 Gene set enrichment analysis

The Pearson correlation coefficients between IER3 and all other genes were calculated using the cor.test function in R. Following this calculation, all genes were ranked in descending order according to their correlation with the target gene. This ranked gene list was then utilized for GSEA to determine whether gene sets exhibiting a strong correlation with the target gene are enriched in specific biological pathways or functional modules. The primary objective of this analysis was to identify the gene sets that demonstrated significant correlations with the target gene and to elucidate the biological implications of these gene sets.

### 2.10 Construction of protein-protein interaction network

To further elucidate the functions and mechanisms of IER3 in biological processes associated with DM, this study utilized the STRING network data platform (https://string-db.org) to identify protein associations and construct a PPI network. By establishing a specified required confidence threshold of 0.400, we ensured that only high-confidence interactions were included in the network, thereby facilitating the identification of key proteins closely related to the function of IER3. The establishment of this network enhances our understanding of the molecular mechanisms underlying the role of IER3 in DM, as well as the associated signaling pathways and biological processes in which it may be involved. Through this systematic approach, we are able to delineate the critical role of IER3 in the pathophysiology of DM and propose potential molecular targets for future therapeutic strategies. To explore the correlations between IER3 and key genes in the PI3K/Akt and MAPK signaling pathways in DM, we utilized gene expression data from public databases. We identified core genes in the PI3K/Akt pathway, including PIK3CA, PIK3CB, PIK3CD, PIK3R1, AKT1, AKT2, and AKT3, as well as key genes in the MAPK pathway, such as MAPK3, MAPK8, MAPK9, MAPK14, MAP2K1, MAP2K2, and MAP3K4. Following this, we performed a correlation analysis to assess the expression relationships between IER3 and these genes in DM samples. The results were visualized using a heat map to facilitate interpretation of the correlations.

### 2.11 Immuno-infiltration analysis

To attain a deeper insight into the cellular composition and functional alterations within the immune system in the context of DM, this study employed the CIBERSORT algorithm for a comprehensive analysis of immune cell infiltration. CIBERSORT is a deconvolution algorithm that leverages gene expression data to identify the relative abundances of 22 distinct immune cell types, estimating their proportions in heterogeneous cell samples based on a training set derived from established gene expression profiles characteristic of known immune cells (24). The “CIBERSORT” package was employed in our analysis to further elucidate the differences in immune cell proportions between DM patients and healthy control groups, as well as to explore potential correlations between these variations and the immune responses and inflammatory processes associated with DM.

To effectively present the analysis results visually, we applied R packages such as “ggplot2,” “corrplot,” and “vioplot” to effectively illustrate the distribution and interrelationships of various immune cell types across the two groups. Furthermore, Spearman correlation analysis was conducted to assess the association between immune cells and the candidate biomarker IER3, evaluating the impact of IER3 expression levels on the immune cell ratios. This segment of the research not only deepens our understanding of the role of immune cells in the pathological processes of DM, but also provides empirical support for the potential use of IER3 as a key biomarker. Consequently, it offers new insights and viable targets for the diagnosis, treatment, and prognostic evaluation of DM.

### 2.12 Statistical analysis

Statistical analyses were conducted utilizing R software (version 4.4.1), and the Wilcoxon and T-tests were employed to compare differences between the T2DM group and the control group. A p-value of less than 0.05 was considered statistically significant.

## 3 Results

### 3.1 Identification of DEGs in DM

**Figure 1:**
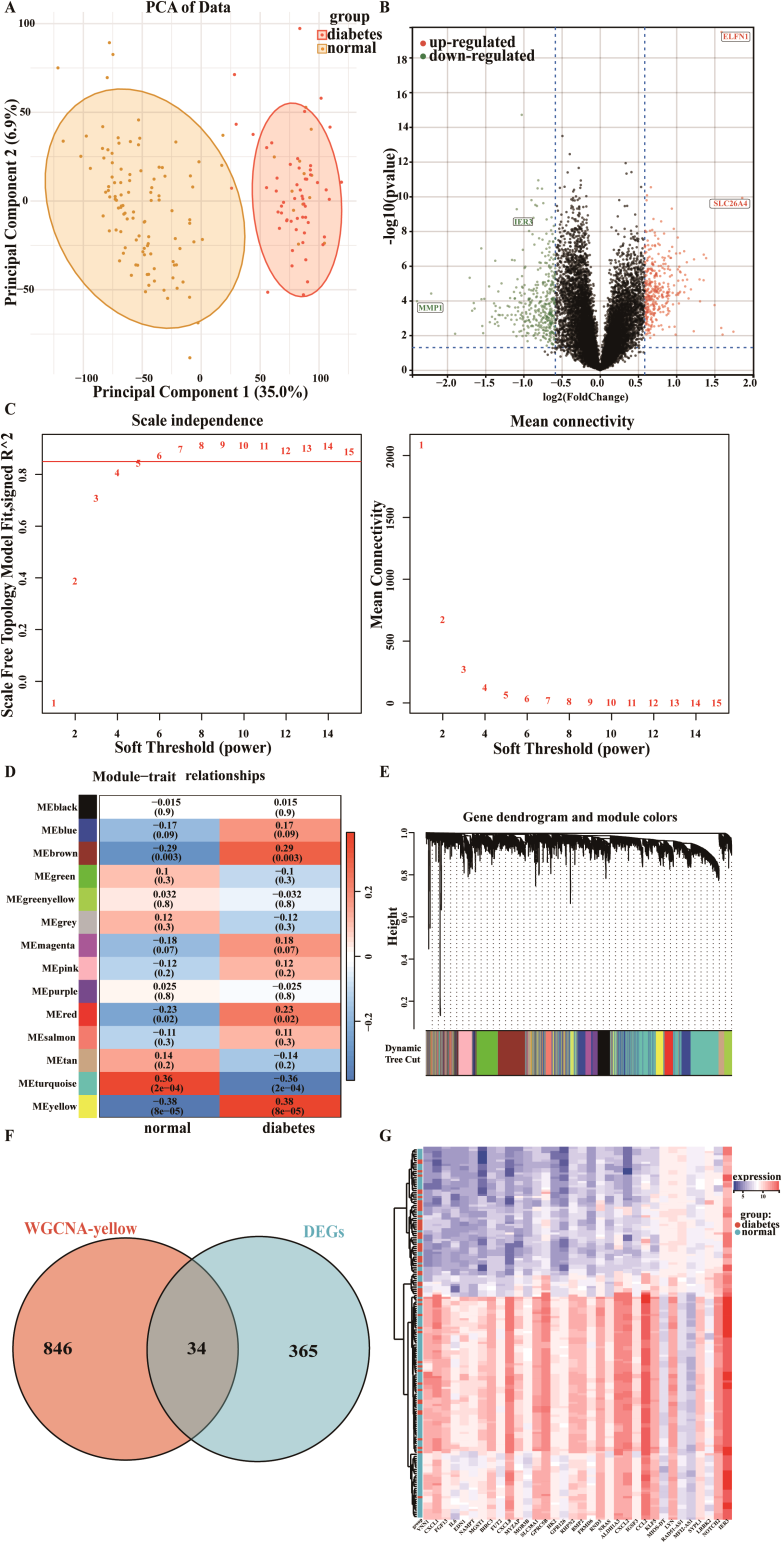
Exploratory analysis of gene expression in DM. **(A)** Principal Component Analysis (PCA). **(B)** A volcano plot illustrating all differentially expressed genes (DEGs). **(C)** Determination of the optimal soft threshold. **(D)** Heatmap depicting the relationship between gene modules and clinical traits. **(E)** Gene cluster tree of co-expressed genes. **(F)** Venn diagram demonstrates the intersection of common genes identified through Weighted Gene Co-expression Network Analysis (WGCNA) and DEGs. **(G)** Cluster heatmap based on all DEGs.

The results of PCA reveal a notable trend of separation between DM patients and the normal population within the PCA space (**Figure 1A**). While some overlapping regions are observed, the overall clustering characteristics of the data points from the two groups demonstrate marked differences. These findings indicate that PCA effectively captures the principal variance patterns within the dataset and partially elucidates the differences between the two groups.

In the GSE76896 dataset, we identified a total of 401 DEGs, comprising 177 upregulated and 224 downregulated genes (**Supplementary Table 1**). The volcano plot (**Figure 1B**) visually illustrates the expression changes and statistical significance of these genes, with orange and green dots representing genes that are significantly upregulated or downregulated in the DM group, respectively. The black dots at the center of the plot indicate genes with no significant changes in expression. Our results reveal that the expression of the IER3 gene is significantly decreased in DM patients compared to the control group, whereas the SLC26A4 and ELFN1 genes exhibit significant upregulation. These key DEGs identified in DM lay the groundwork for further functional analysis.

### 3.2 WGCNA and module gene identification in DM

To identify the gene modules most closely associated with DM, we conducted a WGCNA. The optimal soft threshold for GSE76896 was determined to be 6 (**Figure 1C**). A total of 14 distinct modules were then identified, among which the MEyellow module demonstrated the strongest negative correlation with DM (correlation coefficient = -0.38, p = 8e-05) (**Figures 1D, E**) (**Supplementary Table 2**), encompassing 882 genes. We subsequently intersected the DEGs with the genes selected through WGCNA, resulting in a set of 34 shared genes associated with DM (**Figure 1F**) (**Supplementary Table 3**). A clustering heatmap for these 34 DM-related genes was generated using the “ggplot2” and “pheatmap” R packages (**Figure 1G**).

### 3.3 GO enrichment analysis and KEGG pathway analysis

**Figure 2:**
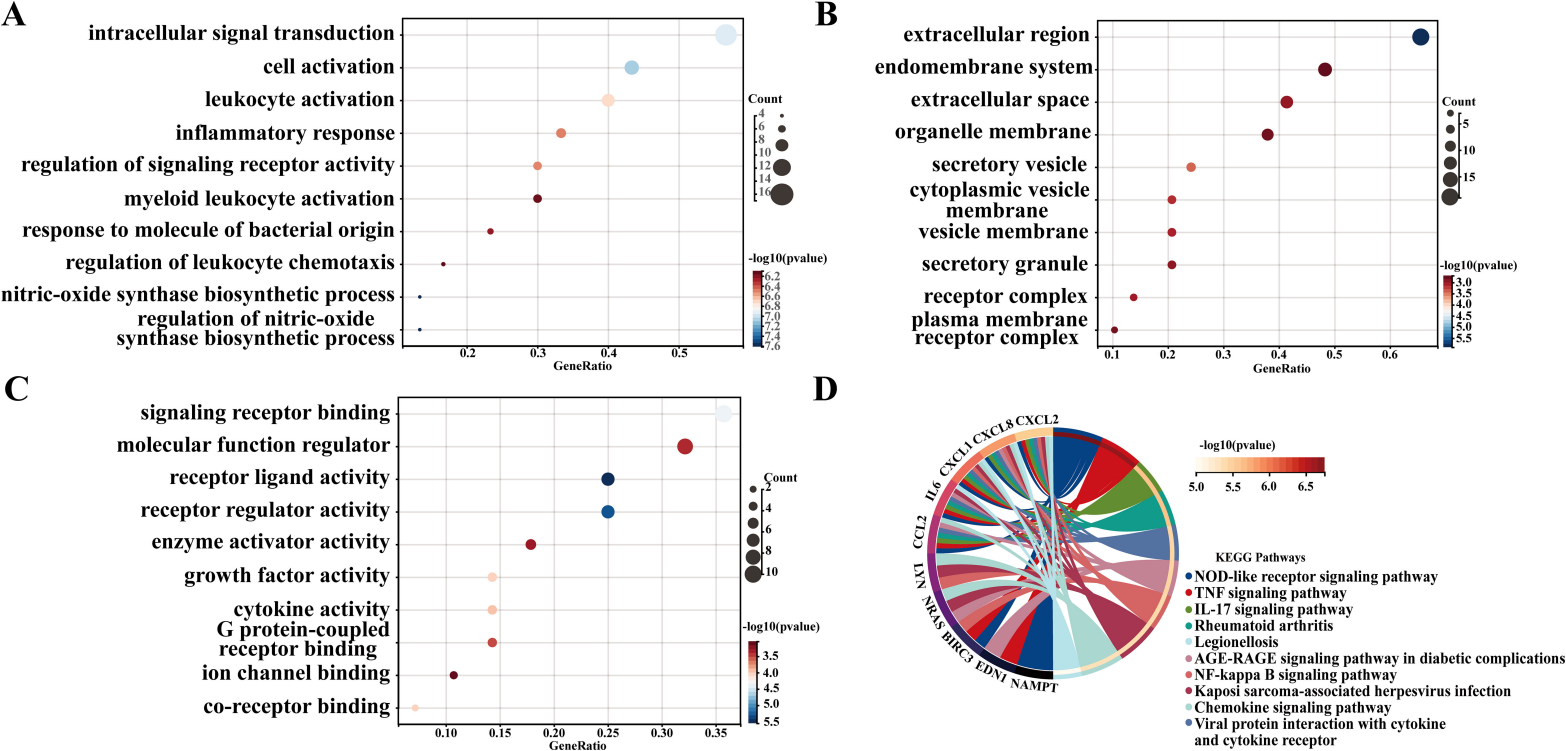
GO and KEGG analyses of diabetes-related genes. **(A–C)** Gene Ontology (GO) categories for Biological Processes (BP), Cellular Components (CC), and Molecular Functions (MF). The top 10 categories of BP, CC and MF are shown. **(D)** Kyoto Encyclopedia of Genes and Genomes (KEGG) enrichment analysis.

To further explore the biological functions of the identified DM-related genes and to uncover potential key signaling pathways involved, we conducted GO enrichment analysis (**Figures 2A–C**) and KEGG pathway analysis (**Figure 2D**). The top ten enriched BPs included intracellular signal transduction, cell activation, leukocyte activation, inflammatory response, regulation of signaling receptor activity, myeloid leukocyte activation, response to molecule of bacterial origin, regulation of leukocyte chemotaxis, nitric-oxide synthase biosynthetic process, and regulation of nitric-oxide synthase biosynthetic process. Notably, the enrichment of nitric oxide synthase biosynthetic process regulation aligns with emerging evidence linking endothelial dysfunction to DM (25). In this context, impaired NO bioavailability contributes to vascular complications (26). The top ten enriched CC were identified as extracellular region, endomembrane system, extracellular space, organelle membrane, secretory vesicle, cytoplasmic vesicle membrane, vesicle membrane, secretory granule, receptor complex, and plasma membrane receptor complex. CC analysis highlighted significant extracellular space and secretory vesicles, indicating dysregulated paracrine signaling. For instance, extracellular vesicles derived from β cells can serve as a medium for intercellular communication within the pancreatic microenvironment in type 1 DM and participate in immune regulation (27). Furthermore, the top ten enriched MF included receptor ligand activity, receptor regulator activity, signaling receptor binding, co-receptor binding, growth factor activity, cytokine activity, G protein-coupled receptor binding, molecular function regulator, enzyme activator activity, and ion channel binding. Additionally, Ion channel binding may be associated with potassium channel mutations that lead to insufficient insulin secretion in response to glucose levels (28). Following this, in terms of KEGG pathways, the top ten pathways identified were the NOD-like receptor signaling pathway, TNF signaling pathway, IL - 17 signaling pathway, Rheumatoid arthritis, Viral protein interaction with cytokine and cytokine receptor, AGE-RAGE signaling pathway in diabetic complications, NF-kappa B signaling pathway, Kaposi sarcoma-associated herpesvirus infection, Chemokine signaling pathway, and Legionellosis. Notably, these findings of the GO classification and KEGG pathway analysis reveal the functional characteristics of DM-related genes at the molecular biological and signaling transduction levels, particularly in relation to immune responses, signal transduction, and metabolic regulation, thereby providing crucial insights into the molecular pathophysiological mechanisms underlying the onset of DM.

### 3.4 Identification of candidate biomarkers for DM through machine learning

**Figure 3:**
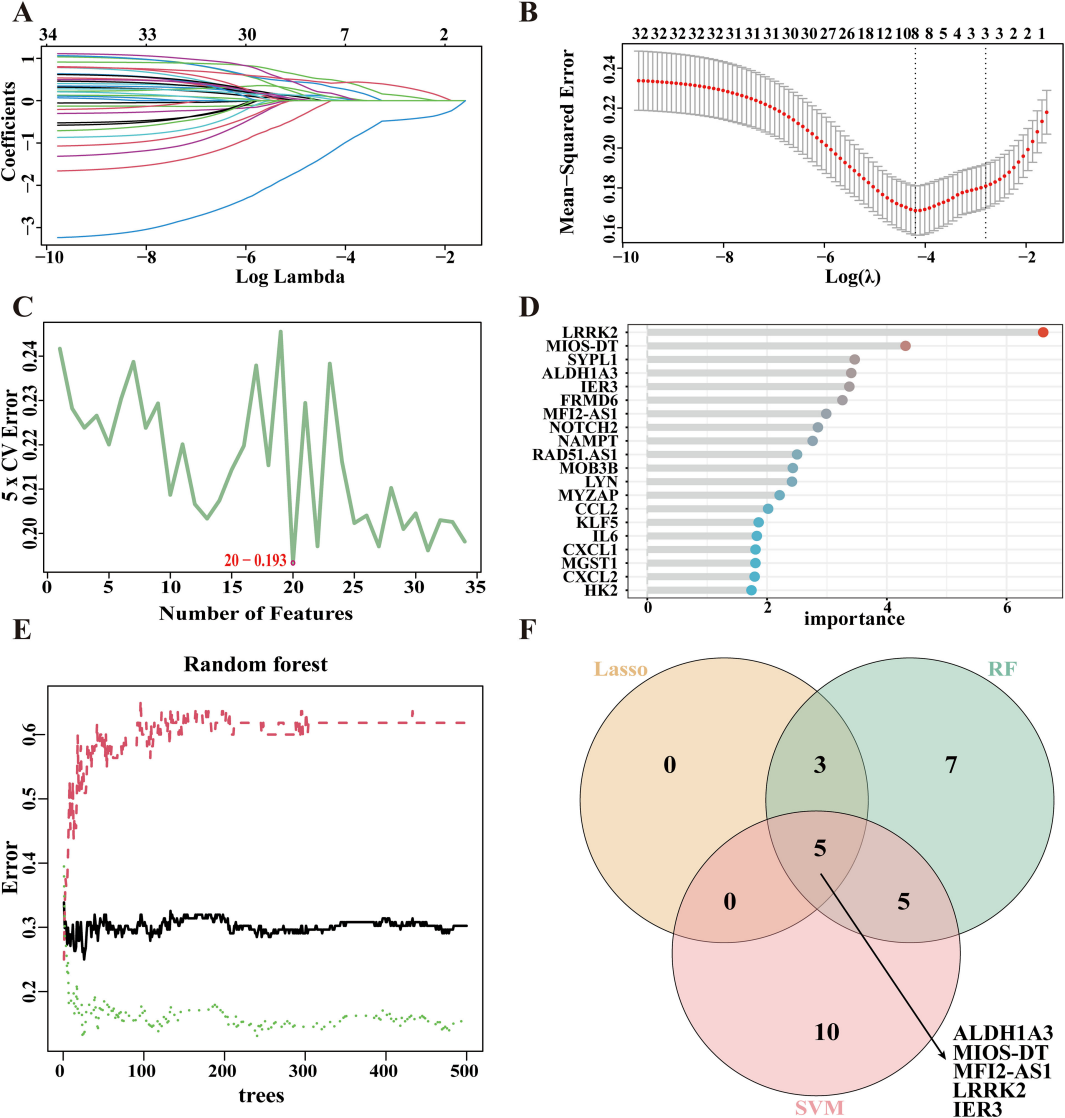
Machine learning in the screening of candidate biomarkers. **(A, B)** Based on the Lasso regression algorithm, 8 genes corresponding to the lowest binominal deviation were identified as the most appropriate for diabetes mellitus (DM) diagnosis. **(C)** The top 20 genes were selected based on Support Vector Machine Recursive Feature Elimination (SVM-RFE) with the lowest error rates and highest accuracy for DM classification. **(D, E)** The top 20 genes were selected and ranked according to the importance scores derived from the random forest algorithm applied to DM. **(F)** A Venn diagram showing the intersected genes identified by the three machine learning algorithms in DM.

To further refine the identification of key genes associated with DM, we identified 34 common genes by intersecting 401 DEGs with 882 genes selected through WGCNA. Subsequently, we utilized three machine learning algorithms to screen for potential candidate biomarkers based on these 34 common genes. In the GSE76896 dataset, the LASSO regression identified eight genes (**Figures 3A, B**), whereas the SVM-RFE algorithm extracted 20 genes with the lowest root mean square error (RMSE) (**Figure 3C**). Additionally, the RF classifier ranked the top 20 genes according to their importance (**Supplementary Table 4**, **Figures 3D, E**). By intersecting the results obtained from these three methods, we ultimately identified five candidate biomarkers for DM, including ALDH1A3, MIOS-DT, MELTF-AS1, LRRK2, and IER3 (**Figure 3F**).

### 3.5 Risk stratification of candidate biomarkers for DM

**Figure 4:**
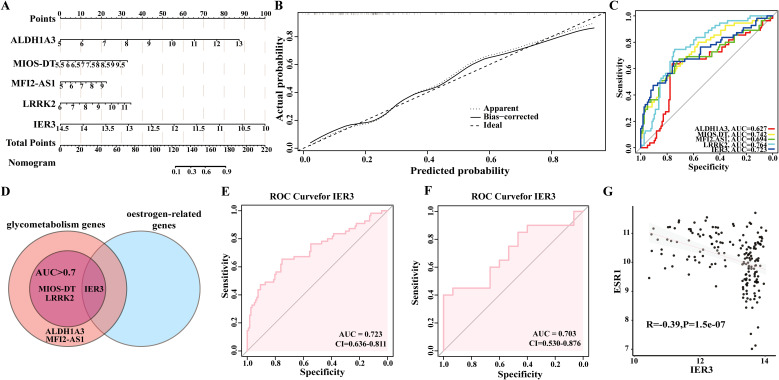
Analysis of IER3 as a candidate biomarker for DM. **(A, B)** Nomogram construction based on five shared genes identified in discovery datasets. **(C)** The Receiver Operating Characteristic (ROC) curve for the shared genes in discovery datasets. **(D)** IER3 was identified as a candidate biomarker. **(E, F)** The ROC curve of IER3 in the GSE76896 and GSE72377 datasets. **(G)** The correlation between IER3 and ESR1 in diabetes.

We subsequently constructed a nomogram (**Figures 4A, B**) based on the above five identified candidate biomarkers for DM, which translates the relative expression levels of each gene into a specific score ranging from 0 to 100. By aggregating the individual gene scores to obtain a total score, we can effectively evaluate the overall risk of an individual developing DM. Specifically, a higher total score correlates with an increased risk of DM occurrence. This methodology not only furnishes clinicians with a robust tool for risk stratification of patients but also holds significant predictive value for the prognosis of DM.

Furthermore, we evaluated the diagnostic performance of each gene as a biomarker for DM through ROC curve analysis (**Figure 4C**). The resulting AUC values were as follows: ALDH1A3 (AUC: 0.627), MIOS-DT (AUC: 0.742), MELTF-AS1 (AUC: 0.694), and LRRK2 (AUC: 0.764), and IER3 (AUC: 0.723). These findings not only enhance our understanding of the molecular mechanisms underlying the onset of DM but also provide valuable biomarkers for prospective clinical applications in the prevention and treatment of DM.

### 3.6 Significance of estrogen-related gene IER3 as a diagnostic and prognostic marker for DM

In this study, we identified the estrogen-related genes and intersected them with the five candidate genes for DM that previously identified through machine learning techniques. We specifically focused on genes exhibiting an AUC value of ≥0.7, ultimately determining IER3 as a key biomarker for DM (**Figures 4D, E**). ROC curve analysis revealed that IER3 achieved an AUC value of 0.723, with a 95% confidence interval ranging from 0.636 to 0.811. This finding suggests that IER3 demonstrates both accurate and satisfactory diagnostic and prognostic value for DM. Furthermore, the ROC curve revealed sensitivity and specificity values for IER3 of 0.8205 and 0.7636, respectively. These performance metrics further underscore the significant role of IER3 as an effective biomarker for DM, highlighting its potential clinical utility. To further evaluate the accuracy of the candidate biomarkers, we employed the GSE72377 dataset for verification and ROC curve analysis revealed that IER3 exhibits significant diagnostic value, with an AUC value of 0.703 (**Figure 4F**). As shown in **Figure 4G**, a significant negative correlation was observed between the expression levels of IER3 and ESR1 (R =- 0.39, P = 1.5e-07). The trend line, along with the 95% confidence interval, is represented in gray. These findings offer evidence suggesting a potential association between IER3 and estrogen signaling pathways.

### 3.7 PPI network analysis of IER3 in DM

**Figure 5:**
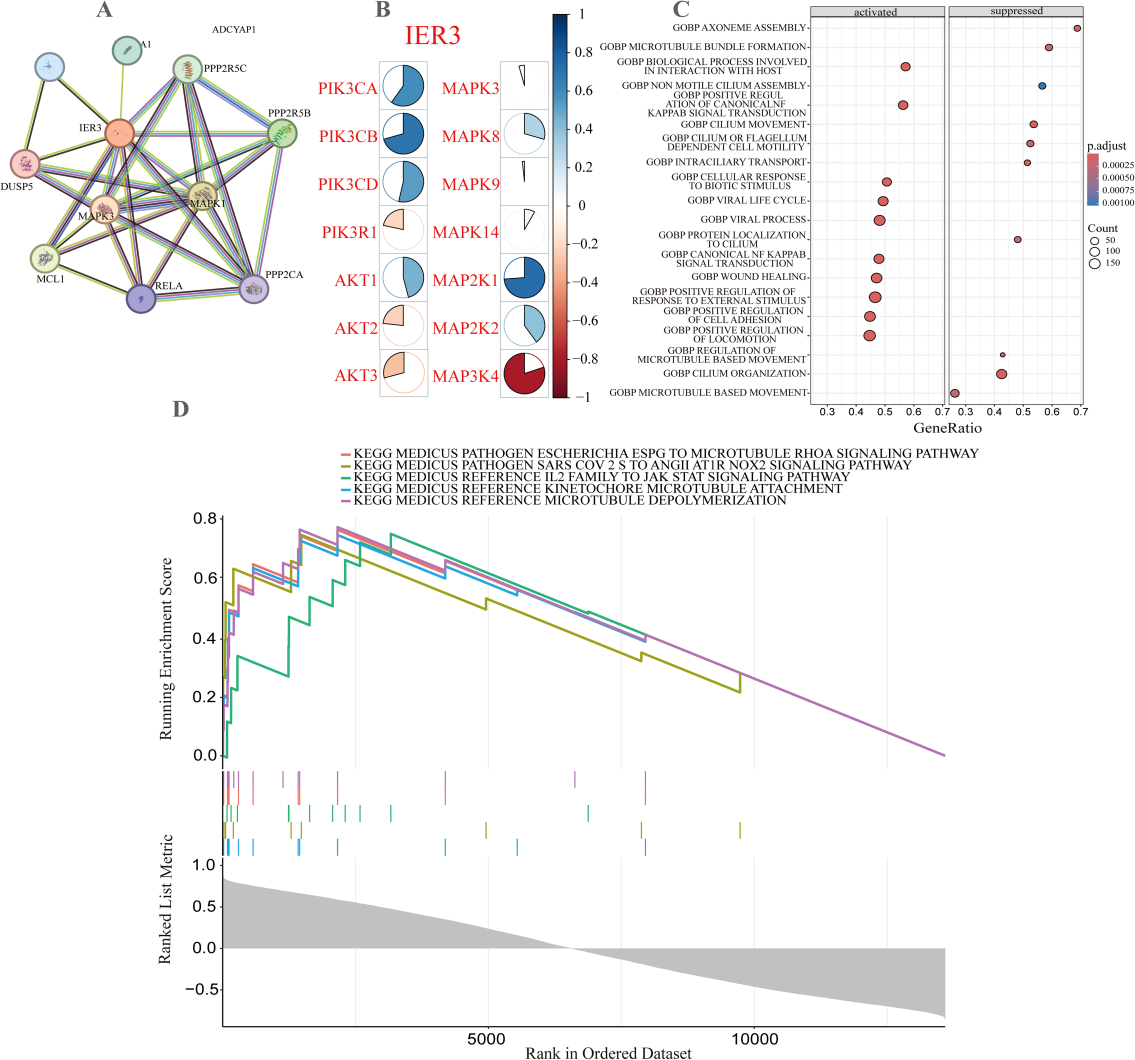
PPI network and functional enrichment analysis of IER3 in DM. **(A)** The network of interacting genes associated with IER3. The circles represent the query proteins and their corresponding first shell interactors in the network. The color and number of edges indicate the source and quantity of supporting evidence, respectively. **(B)** Correlation heatmap of IER3 with genes involved in the PI3K/Akt and MAPK signaling pathways. **(C)** Gene Set Enrichment Analysis (GSEA) illustrating pathway enrichment across the ordered gene dataset. **(D)** KEGG analysis of the activated and repressed biological processes.

PPI network analysis serves as a crucial tool for elucidating gene functions and their biological roles. To further investigate the role of the IER3 gene in DM more comprehensively, we constructed a PPI network centered on IER3 utilizing the STRING database (**Figure 5A**). This network not only illustrates the direct and indirect interactions between IER3 and its interactive genes but also offers valuable insights into the strength and sources of evidence supporting these interactions.

Using this high-throughput analytical approach, we successfully identified the protein nodes that are closely associated with IER3, specifically DUSP5, PHLDA1, ADCYAP1, PPP2R5C, PPP2R5B, MAPK1, MCL1, MAPK3, RELA, and PPP2CA. These protein nodes are depicted in the network with varying colors and line styles, effectively illustrating the positioning of IER3 within the network and its potential influence on other biomolecules. The identification of these interacting proteins provides valuable insights into the potential roles of IER3 in the pathological processes of DM, thereby enhancing our understanding of the molecular pathways through which IER3 is involved in the progression of DM. The heatmap illustrates significant correlations between IER3 and genes involved in the PI3K/Akt and MAPK signaling pathways associated with diabetes (**Figure 5B**). In the PI3K/Akt pathway, both PIK3CA and PIK3CB exhibit strong positive correlations with IER3. Within the MAPK pathway, MAP2K1 shows a positive correlation with IER3, while MAP3K4 reveals a negative correlation. These findings suggest that IER3 may play a role in the pathogenesis of diabetes through its interactions with specific genes in these pathways.

### 3.8 Functional enrichment of IER3

To further elucidate the functions of genes and their underlying biological mechanisms, we conducted GSEA enrichment analysis to identify differentially expressed genes between the low and high expression groups of IER3 (**Figure 5C**). In the GO enrichment analysis, the most significantly activated biological process identified was axoneme assembly, followed by processes such as microtubule bundle formation, host interaction, non-motile cilium assembly, and positive regulation of canonical NF-κB signaling. Additionally, the top five KEGG pathways identified included the Escherichia ESPG to microtubule RHOA signaling pathway, the SARS-CoV-2 spike protein to ANGII/AT1R/NOX2 signaling pathway, the IL - 2-JAK-STAT signaling pathway, kinetochore microtubule attachment, and microtubule depolymerization (**Figure 5D**). These findings indicate that IER3 may be involved in various complex biological processes related to DM, including infection, cardiovascular diseases, immune regulation, cellular dynamics, and cytoskeletal remodeling.

### 3.9 Immune cell infiltration analysis

**Figure 6:**
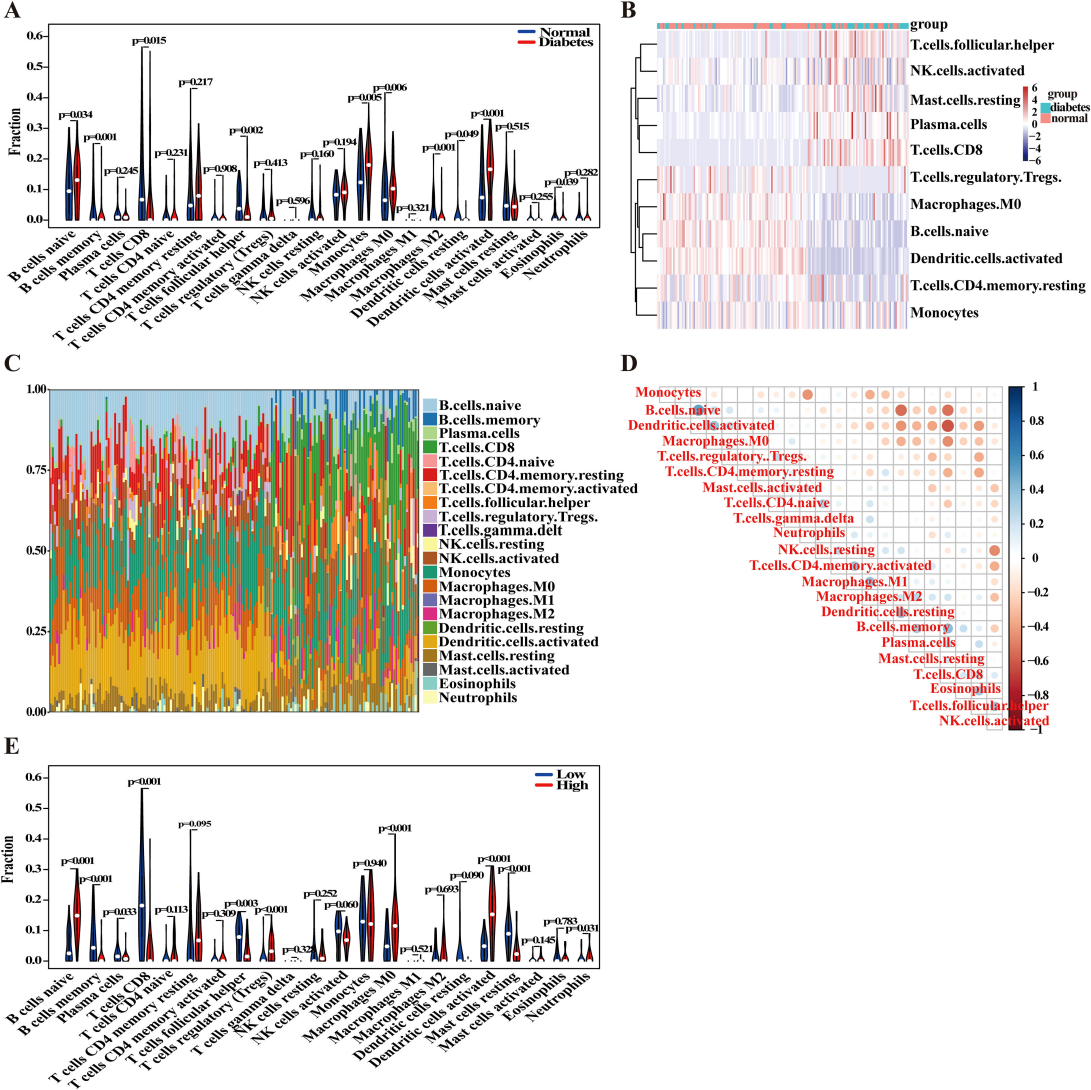
Analysis of immune cell infiltration. **(A)** The boxplot comparing the proportion of immune cells between DM and control groups. **(B)** Comparative heatmap depicting immune cell gene expression in DM and control groups. **(C)** The bar plot visualizing the proportion of infiltrating immune cells in different samples. **(D)** Correlation heatmap representing associations between various immune cell types. **(E)** The boxplot comparing the proportions of immune cells in high and low IER3 expression groups.

In this study, we conducted a comprehensive analysis of the cellular composition and functional alterations of the immune system in the context of DM. Utilizing the CIBERSORT algorithm, we performed a detailed comparison of immune cell proportions between the DM group and normal controls (**Figure 6A**). Our findings revealed significant differences in the proportions of various immune cell types between the two groups, which may be closely related to the immune response and inflammatory processes associated with DM. Specifically, the proportions of naive B cells, monocytes, M0 macrophages, and activated dendritic cells were significantly elevated in the DM group compared to the control group. Conversely, the proportions of CD8+ T cells and follicular helper T cells markedly decreased in the DM group. To further investigate the activation states of different immune cells in DM, we constructed heatmaps to analyze the gene expression patterns of various cell types (**Figure 6B**) and visualized the proportions of different immune cell types (**Figure 6C**). The results indicated that the distribution of multiple immune cell types in the DM group differed significantly from that of the normal group, thereby reinforcing the role of immune cells in the pathology of DM. An in-depth analysis through correlation heatmaps illustrated the relationships among various immune cell types, revealing a notably high degree of similarity between different T cell subtypes, such as resting CD4 memory T cells and CD8+ T cells (**Figure 6D**). This observation suggests potential functional synergy among these cells. Overall, the correlation analyses underscore the intricate interactions and regulatory mechanisms of diverse immune cells in the context of DM.

To further explore the influence of IER3 on the proportions of the aforementioned immune cells, we stratified the DM group into two subgroups based on high and low expression levels of IER3 (**Figure 6E**). The results revealed that the proportions of naive B cells, regulatory T cells (Tregs), activated dendritic cells, and neutrophils were significantly elevated in the high IER3 expression group compared to those in the low expression group. Conversely, the proportions of CD8+ T cells and follicular helper T cells were markedly reduced in the high IER3 expression group. Notably, consistent trends were observed in the proportions of naive B cells, CD8+ T cells, follicular helper T cells, and activated dendritic cells across both comparisons of immune cell proportions. These findings strongly suggest that IER3 plays a pivotal role in modulating the immune microenvironment, thereby influencing the progression of DM. The elevated expression of IER3 appears to be associated with enhanced immune cell activation and increased inflammatory responses. This segment of the research not only underscores the significance of immune cells in the pathological processes of DM but also provides additional empirical evidence for IER3 as a potential biomarker, opening new avenues for the diagnosis, treatment, and prognostic evaluation of DM.

Collectively, these results demonstrate significant changes in immune cell composition under DM conditions, and IER3 is not only closely correlated with variations in immune cell proportions but also plays a crucial role in the immunoregulatory mechanisms underlying DM. These findings underscore the considerable research value of IER3 in elucidating the immunological basis of DM and suggest its potential as a biomarker for future therapeutic strategies.

## 4 Discussion

DM is characterized as a complex metabolic disorder syndrome, distinguished by hyperglycemia, insulin resistance, and hyperinsulinemia, making it one of the most prevalent chronic metabolic diseases globally (29). This condition significantly affects individuals’ overall quality of life (30). Estrogen plays a crucial protective role in the pathogenesis of DM by enhancing both insulin sensitivity and secretion, thereby contributing to the maintenance of stable blood glucose levels (31). Nevertheless, postmenopausal women frequently experience increased insulin resistance and a heightened risk of developing DM due to declining estrogen levels (11, 32). Research has demonstrated that estrogen can regulate pancreatic beta cell function (33), facilitate glucose uptake and utilization, and reduce cellular apoptosis, all of which are critical for preventing and controlling the onset and progression of DM (34). This regulatory effect of estrogen is particularly vital for women’s health.

This study employs a comprehensive approach that integrates bioinformatics methods with machine learning techniques to explore the shared genes and associated signaling pathways related to DM and estrogen. It specifically highlights the potential role of the estrogen-related gene IER3 in DM. The findings reveal a significant downregulation of IER3 in DM patients, and it appears to affect the progression of DM through the regulation of glucose metabolism, immune responses, and inflammatory pathways, suggesting that IER3 may play a pivotal role in the pathological processes linking DM and estrogen. Furthermore, the construction of a diagnostic ROC curve based on IER3 gene expression demonstrates both accurate and satisfactory diagnostic and prognostic value of IER3 for DM. Notably, the study reveals significant changes in immune cell composition under DM conditions, and IER3 is not only closely correlated with variations in the proportions of various immune cells, but also plays a crucial role in the immunoregulatory mechanisms underlying DM. Through an in-depth analysis of IER3 and its associated signaling pathways, this research underscores the unique value of the estrogen-related gene IER3 as a potential biomarker and therapeutic target for DM. Collectively, our study lays the groundwork for future investigations into the molecular mechanisms underlying the pathogenesis of DM, while also providing more molecular evidence and therapeutic strategies for its diagnosis and treatment.

IER3 plays a crucial role in regulating cell apoptosis and the heterogeneity of immune cells (35). Research indicates that macrophages are key contributors to obesity-related inflammation, particularly through the transition of adipose tissue macrophages from alternatively activated macrophages (AAM) to classically activated macrophages (CAM), a process that is significant in the context of obesity-associated inflammation (36, 37). The high expression of IER3 in macrophages may facilitate this transformation, thereby promoting the onset of obesity-related inflammation and enhancing insulin sensitivity in murine models (38). Additionally, IER3 is extensively implicated in vital biological processes such as cell proliferation, differentiation, and apoptosis, with its expression regulated by various transcription factors, including NF-κB, p53, SP1, AP1, vitamin D3 receptor (VD3R), and retinoic acid receptors (RAR/RXR) (39, 40). Furthermore, studies have highlighted the prognostic value of IER3 in several pathological conditions, including pancreatic cancer, hepatocellular carcinoma, and acute kidney injury (41–44).

Estrogen plays a pivotal regulatory role in the onset and progression of DM, particularly among female patients, where fluctuations in estrogen levels may directly affect insulin sensitivity and glucose metabolism (45). This study posits that IER3 may serve as an intermediary between DM and estrogen, thereby establishing a critical connection between the two. The expression of the IER3 gene is modulated by various factors, with estrogen emerging as a significant regulator that may influence the development of DM through its impact on IER3 expression. Furthermore, our findings indicate a significant negative correlation between the expression levels of IER3 and ESR1, suggesting a potential association between IER3 and estrogen signaling pathways. Additionally, studies have shown that IER3 exhibits a dose-dependent response to 17β-estradiol stimulation in MCF - 7 (BUS) cells, with its expression being upregulated in conjunction with cyclin D1 and its mutants (46). These findings collectively underscore the potential regulatory role of estrogen on IER3 and highlight the importance for further investigation into this gene and its associated pathways. Such investigations will enhance our understanding of the pathological mechanisms underlying DM and may offer novel therapeutic targets for clinical intervention.

In addition to its involvement in glucose metabolism and estrogen levels, the IER3 gene may also participate in the immune regulatory mechanisms associated with DM by modulating immune system functionality. Recently, the interplay between immune responses and DM has garnered significant attention (47). Research has indicated that the chronic inflammatory state characteristic of DM is closely linked to the aberrant activation of immune cells (48, 49). The dysregulation of immune cell subset proportions constitutes a critical pathological hallmark within the immune microenvironment of DM. Significant elevations in the proportions of naive B cells, monocytes, M0 macrophages, and activated dendritic cells (DCs) were observed in the DM patients. Research has demonstrated that in insulin-dependent DM, activated DCs play a crucial role in autoimmune pathogenesis by presenting β-cell-derived autoantigens to naive autoreactive Th0 lymphocytes (50). This antigen presentation facilitates the differentiation of Th0 cells into pro-inflammatory effector T cells, which subsequently initiate β-cell apoptosis through cytotoxic mechanisms. The resulting impairment of insulin biosynthesis in pancreatic islets constitutes a key pathogenic mechanism in disease progression, with DC-mediated antigen presentation serving as a pivotal initiating event in the autoimmune destruction of β-cells. Monocytes also contribute significantly to the vascular complications associated with DM. In the diabetic environment, monocytes are recruited to the vascular wall, leading to a rapid release of inflammatory cytokines such as IL - 1β and TNF-α, which accelerate the progression of atherosclerotic lesions and plaque instability (51). Our study revealed significant reductions in CD8^+^ T cells and follicular helper T cells among DM patients. As primary cytotoxic lymphocytes, the depletion of CD8^+^ T cells may be linked to functional exhaustion characterized by PD - 1 upregulation and metabolic dysregulation manifested by glycolytic inhibition and mitochondrial dysfunction. Consequently, this depletion diminishes their capacity to eliminate aberrant cells in target tissues (52, 53). Furthermore, follicular helper T cells play a pivotal role in maintaining immune tolerance and regulating B-cell antibody production, with their diminished frequency potentially predisposing to aberrant humoral immune responses (54). This pathological process may exacerbate β-cell dysfunction through disrupting local T-B cell interactions within pancreatic islets and impairing antigen-specific immunomodulation. Collectively, the imbalance of immune cell repertoires in DM is not merely a passive epiphenomenon, it likely drives metabolic derangements, islet dysfunction, and chronic inflammation via mechanisms involving immunometabolic decoupling, dysregulated cytokine release, and impaired local immune regulation. These findings underscore the centrality of immune cell dyshomeostasis in elucidating the pathophysiological progression of DM. Our findings indicate a strong correlation between IER3 expression and alterations in the proportions of immune cells, particularly in patients with DM, suggesting that dysregulation of the immune system may exacerbate the progression of DM by influencing the activation states of immune cells. Therefore, IER3 may be pivotal in regulating the chronic inflammatory response associated with DM through its impact on immune system functionality. Several studies have highlighted the significant role of IER3 in immune cells, potentially modulating the release of cytokines, the activation of immune cells, and their migration, thereby affecting systemic inflammatory responses (55).

This study elucidates the potential biological and immunological significance of IER3 in DM by employing an integrated approach that combines bioinformatics and machine learning techniques. However, it remains in its preliminary stages and has certain limitations. The molecular mechanisms that link IER3 to estrogen signaling pathways, specifically the PI3K/Akt and MAPK cascades, along with their interactions with immune regulation, require experimental validation. Moreover, the causal relationship between IER3 downregulation and the progression of DM necessitates verification through longitudinal studies and interventional models. Future research should strive to diversify data sources by incorporating a wide range of sample data from DM patients across various ethnicities and regions, thereby enhancing the reliability and generalizability of the findings. Furthermore, it is essential to clarify the relationship between IER3 and different types of DM, such as type 1 diabetes and gestational diabetes, in order to further deepen and broaden the scope of the research. Therefore, such future efforts have the potential to substantially enhance the applicability of IER3 in the treatment of DM.

## 5 Conclusion

In this study, we conducted a thorough investigation focusing on the role of the estrogen-related gene IER3 in the context of DM. Our findings reveal a significant downregulation of IER3 in DM patients, with an AUC value of 0.723 on the diagnostic ROC curve, indicating its considerable diagnostic and prognostic potential for DM. Furthermore, IER3 acts as a critical link between DM and estrogen, influencing the progression of DM through its regulatory effects on glucose metabolism, immune responses, and inflammatory pathways. Notably, our study uncovers significant alterations in immune cell composition under DM conditions. IER3 is not only closely correlated with variations in the proportions of diverse immune cell types but also plays a crucial role in the immunoregulatory mechanisms underlying DM. Through an in-depth analysis of IER3 and its associated signaling pathways, this research emphasizes the unique value of the estrogen-related gene IER3 as a potential biomarker and therapeutic target for DM.

Conclusively, these findings offer valuable insights into the biological and immunological significance of IER3. Monitoring its expression could facilitate the identification of high-risk populations, and its significance in the early diagnosis and prognostic evaluation of DM should not be underestimated. Consequently, extensive research on IER3 and its related signaling pathways opens new avenues for the development of innovative diagnostic tools and therapeutic strategies for the prevention and management of DM. Future investigations should explore the modulation of IER3 expression through pharmacological or gene-editing techniques, aiming to establish new treatment strategies for DM and provide essential evidence for personalized therapy. We anticipate that further exploration in this field will facilitate advancements in relevant technologies and their practical applications, ultimately enhancing the quality of life and health outcomes for individuals affected by DM.

## Data Availability

The datasets presented in this study can be found in online repositories. The names of the repository/repositories and accession number(s) can be found in the article/**Supplementary Material**.
